# Evaluating national guidelines for monitoring early growth using routinely collected data in Bergen, Norway

**DOI:** 10.1177/14034948231187513

**Published:** 2023-07-27

**Authors:** Melissa R. Balthasar, Mathieu Roelants, Bente Brannsether-Ellingsen, Kristine M. Stangenes, Maria C. Magnus, Siri E. Håberg, Simon N. Øverland, Pétur B. Júlíusson

**Affiliations:** 1 Department of Paediatric and Adolescent Medicine, Stavanger University Hospital, Norway; 2 Department of Clinical Science, University of Bergen, Norway; 3 Department of Public Health and Primary Care, KU Leuven, University of Leuven, Belgium; 4 Department of Health Registry and Development, Norwegian Institute of Public Health, Norway; 5 Centre for Fertility and Health, Norwegian Institute of Public Health, Norway; 6 Section for Health Care Collaboration, Haukeland University Hospital, Norway; 7 Department of Paediatric and Adolescent Medicine, Haukeland University Hospital, Norway

**Keywords:** Growth monitoring, percentile crossing, growth reference, growth standards, national guidelines, electronic health records, percentile curve, Norway

## Abstract

**Aims:**

The overarching aim of this study was to evaluate the Norwegian guidelines for growth monitoring using routinely collected data from healthy children up to five years of age. We analysed criteria for both status (size for age) and change (centile crossing) in growth.

**Methods:**

Longitudinal data were obtained from the electronic health record (EHR) at the well-baby clinic for 2130 children included in the Bergen growth study 1 (BGS1). Measurements of length, weight, weight-for-length, body mass index (BMI) and head circumference were converted to *z*-scores and compared with the World Health Organization (WHO) growth standards and the national growth reference.

**Results:**

Using the WHO growth standard, the proportion of children above +2SD was generally higher than the expected 2.3% for all traits at birth and for length at all ages. Crossing percentile channels was common during the first two years of life, particularly for length/height. By the age of five years, 37.9% of the children had been identified for follow-up regarding length/height, 33% for head circumference and 13.6% for high weight-for-length/BMI.

**Conclusions:**

**The proportion of children beyond the normal limits of the charts is higher than expected, and a surprisingly large number of children were identified for rules concerning length or growth in head circumference. This suggests the need for a revision of the current guidelines for growth monitoring in Norway.**

## Introduction

Growth monitoring is a cornerstone of preventive child health care. It provides an overall appraisal of children’s thriving and can signal conditions that affect growth and warrant further care [1]. For this reason, aberrant growth is commonly used as an indicator for further clinical evaluation or referral. Population-specific reference data on child growth usually increase the accuracy when screening for abnormal growth [2–5].

Individual measurements are typically compared to size-for-age percentile charts or standard devia-tion (SD) charts for consideration of normality [6]. Alternatively, measurements can be expressed as *z*-scores (SD scores), which quantifies the degree of deviation from the reference population mean. A *z*-score between −2 and +2 SD covers approximately 95% of the reference population. A common growth chart format is to display the distribution of selected body measurements using seven equally spaced centile lines which corresponds to the age-specific percentiles (p): 3, 10, 25, 50, 75, 90 and 97. The space between these percentiles, the ‘percentile channel’, corresponds to a *z*-score difference of approximately 0.67 SD scores, while the extremes (p3 and p97) are approximately comparable to a z-score of −2 and +2, respectively. The design of the World Health Organization (WHO) growth standards is somewhat different, as it shows five centiles spaced with 1 SD.

Crossing one or two percentile channels (0.67 or 1.34 SD scores) for length and particularly weight during the first two to three years of life is considered normal and may occur frequently during the first six months [7,8]. An upward change of more than 0.67 SD scores in length or weight can reflect catch-up growth [9,10], while a downward change moving to a lower percentile on the growth chart can be evident as catch-down growth [11]. In cases of catch-down growth, possible underlying conditions such as malnutrition, endocrine, genetic or chronic diseases should be considered [11]. In a similar manner, concerning head circumference, both a downward and upward shift may mirror serious conditions that need prompt attention [12]. While the expected prevalence of being small (light) or tall (heavy) at a given age can be deduced from the reference curve (e.g. 3% are expected to be below the third percentile of a matching reference), this is not true for percentile crossing. The literature on prevalence of channel crossing is almost dated [7,13] and lacking for the population of Norwegian children.

The Norwegian Directorate of Health recommended using the WHO growth standards for monitoring length/height and weight from birth to five years in primary health care, although Norwegian children were found to be taller and heavier compared to the WHO standards [2,14,15]. For the assessment of head circumference, the national Norwegian growth references based on the Bergen Growth Study 1 (BGS1) were recommended, as Norwegian children were found to have clinically significant larger heads [2]. Effective growth monitoring to detect health conditions that need attention depends on both the cut-off criteria and the growth charts in use [16,17]. The national guidelines on growth monitoring assist health-care workers to interpret growth status and detect children for further action (i.e. identifying for follow-up or referral to specialist health care) [14]. These guidelines have been developed by an expert panel group and are largely based on established practice and clinical experience. However, they are lacking data on the prevalence of healthy children being identified.

The overarching aim of the present study was to evaluate the Norwegian guidelines for growth monitoring using routinely collected data with a cohort of healthy children. The guidelines include rules based on status (size for age), using the 3rd and 97th percentile as cut-off, and rules based on changes in percentile position on the growth chart. We therefore assessed the proportion of 0- to 5-year-old children beyond the common reference percentile limits using the WHO growth standards or the national growth reference as recommended. Second, we estimated how often crossing of percentile channels occurred between scheduled contacts at the well-baby clinic. Lastly, we applied age-appropriate rules to identify children for further follow-up or referral according to the guidelines to the data from each child in order to estimate the impact of the guidelines as a whole.

## Methods

### Study population

The national growth charts used in Norway were developed from the BGS1, which is a cross-sectional study of growth in 8299 children aged 0–19 years recruited in 2003–2006 [18,19]. The present analysis is based on a subsample of 1806 children from the BGS1 for whom consent was obtained to retrieve routine measurements from the well-baby clinics. Longitudinal data of length/height, weight and head circumference from birth to five years of age were retrieved retrospectively from the electronic health record (EHR) Visma HsPro (Oslo, Norway) in 2011. While 64% of parents consented, data within target age range were only retrieved for 2130 children due to the gradual introduction of the EHR and due to children moving to/from other municipalities. Data on gestational age at birth, nationality, health and medical conditions were obtained from a parental questionnaire when the child was enrolled in the BGS1. As our aim was to describe a normal and healthy child population, the present analysis included term-born (37–42 weeks) singletons with a birth weight ⩾2500 g with no medical condition known to affect growth (Supplemental Figure S1). This resulted in an eligible sample of 1806 children with one or more measurements between birth and five years of age. Characteristics of the cohort in this study are presented in Table I.

**Table I.** Characteristics of the children included in the analysis (*N*=1806).

Characteristic	*N*=1806
Sex	
Boys, *n* (%)	952 (53)
Girls, *n* (%)	854 (47)
Ethnicity/country of origin, *n*	
Nordic^a^	1639
Europe	79
South America	13
Asia	56
Africa	14
Others	5
Birth weight (g), *M* (*SD*)	3700 (500)
Birth length (cm), *M* (*SD*)	50.8 (2.1)
Head circumference at birth (cm), *M* (*SD*)	35.5 (1.8)

All children were enrolled in the Bergen growth study 1 between 2003 and 2006, and routine measurements between birth and five years of age were retrieved in 2011.

a Parents originating from the Nordic countries (Norway, Denmark, Sweden, Finland, Island).

At the well-baby clinics, children were measured according to national guidelines using a standardised technique and equipment. Length was measured up to two years of age in the supine position using a board with fixed headplate. From two years of age, height was measured in the standing position using a wall-mounted stadiometer. Weight was measured undressed using a baby scale for infants. From the age of 2 onwards, the child was measured wearing light clothing using a bathroom scale. The body mass index BMI was calculated as the weight in kilograms divided by the square of the length/height in metres (kg/m^2^). Head circumference was measured as the maximum occipital frontal circumference with a flexible, non-stretchable tape. Data on head circumference were limited to the first 12 months because routine measurements are not recommended after that age. Available data after this age may therefore include a relatively large proportion of children with suspected aberrant growth.

Scheduled contacts were grouped by the target ages of six weeks, 3, 6, 12 and 15 months and two and four years (Supplemental Figure S2). Visits two weeks before and four weeks after the target age were included, as all children do not visit the well-baby clinic at the exact target age. If a child was measured twice in that age interval, the measurement nearest to the target age was used. Consultations at 5 and 10 months of age were not included because of their proximity to other consultations.

### Data analysis

Measurements of length, weight, weight-for-length and head circumference were converted to *z*-scores using either the national growth reference [19] or WHO growth standards [20]. The proportion of children below −2 SD or above +2 SD for length/height, weight and head circumference was calculated at each target age. Assuming a Gaussian distribution of *z*-scores, the expected prevalence of children more than two standard deviations from the mean is 2.3% on either side of the normal distribution. BMI cut-offs from the International Obesity Task Force (IOTF) were used to determine overweight and obesity [21].

Percentile crossing for all traits was determined by subtracting the *z*-scores of previous measurements from the current one (e.g. the *z*-score at three months minus the *z*-score at six weeks). The Norwegian guidelines advocate growth charts with a seven-centile format corresponding to a bandwidth of 0.67 SD. Therefore, a change in *z*-score of 0.67 SD scores or more corresponds to the crossing of one or more growth channel(s), and a change of 1.34 SD scores or more corresponds with crossing of two or more growth channels.

The Norwegian national guidelines to monitor growth in infants and children were evaluated by applying the guidelines’ criteria for follow-up and referral (Supplemental Table SII). According to the guidelines, the WHO growth standards are recommended for monitoring length and weight, and the national growth references are recommended for monitoring head circumference.

Follow-up is advised when weight-for-length is >97th percentile (+2SD), but this does not apply to children who are fully breastfed. The mode of infant feeding was not available for all children. Thus, we analysed weight-for-length from six months of age onwards. Differences in early weight development between exclusively breastfed and formula-fed children have been previously documented [2]. To estimate the number of children who would be identified for follow-up or referral according to the guidelines, we only included children who were not already identified on a previous occasion. This reflects more closely the reality under the assumption that children are only referred once for the same or similar problem. Referral rules based on clinical findings are not included in the analysis, as we did not have this information.

Data were analysed in R v3.5 (R Development Core Team, Vienna, Austria) and IBM SPSS Statistics for Windows v26 (IBM Corp., Armonk, NY).

## Results

### Children outside the reference interval

When using the WHO standard (Table II), the proportion of children in Norway with a length and weight that exceeded +2 SD was >2.3%, while the proportion below −2 SD was lower, as previously described [2]. The discrepancy was larger at birth (for all traits) and throughout the whole age range for length/height, although the distribution of length tended to converge towards WHO standards by the age of five years. Differences after birth were generally smaller for weight and weight-for-length.

**Table II.** Prevalence of children below −2SD or above +2SD at selected ages from birth to five years when using the WHO growth standards for length/height and weight and the national growth reference for head circumference.

Age	Length/height-for-age		Weight-for-age		Weight-for-length		Head circumference-for-age^a^	
	*n*	Below −2/above +2 SD (%)	*n*	Below −2/above +2 SD (%)	*n*	Below −2/above +2 SD (%)	*n*	Below −2/above +2 SD (%)
Birth	1793	1.2/11.6	1805	0.0/7.9	1788	1.6/5.3	1535	1.3/3.6
6 weeks	846	0.8/7.1	1100	0.5/2.5	842	3.1/2.3	993	0.3/2.4
3 months	1116	0.6/8.2	1136	0.8/3.1	1113	3.1/0.8	1100	0.4/2.3
6 months	1110	0.4/11.1	1123	0.6/4.4	1108	1.3/2.1	1101	0.6/4.6
12 months	1192	0.7/8.6	1208	0.3/3.9	1190	0.4/2.9	1150	0.8/7.7
15 months	1121	1.0/6.6	1130	0.3/4.2	1111	0.2/4.0		
24 months	1363	0.9/3.1	1383	0.4/3.4	1352	0.4/5.8		
4 years	1540	1.1/2.7	1524	0.2/2.4	1332	0.2/3.2		

The expected prevalence for a matching reference curve is 2.3% below −2 SD and 2.3% above +2 SD.

a Head circumference is systematically recorded up to 12 months of age.

WHO: World Health Organization.

### Crossing of percentile channels

Overall, downward and upward crossing of centile channels was more frequently observed for length than for weight or head circumference, when excluding changes between birth and six weeks. Further, crossing was more frequent before 24 months of age than thereafter.

Using the WHO growth standards (Table III), when excluding changes between birth and six weeks, upward crossing was more prevalent than downward crossing for length and weight during the first six months of life, while the opposite trend was observed after 15 months of age. For weight-for-length, upward crossing was more prevalent than downward crossing between three months and two years. In total, 43% and 41% of the children crossed one or more channels up/down for length and weight, respectively, between the ages of six weeks and one year (Supplemental Table SI).

**Table III.** Prevalence of children crossing one and two percentile channels using changes in *z*-scores based on the WHO growth standards for length/height and weight and on the national growth reference for head circumference.

From	To	*N*	Length/height-for-age		Weight-for-age		Weight-for-length		Head circumference-for-age^*^	
			*n*	<–2/<–1/>1/>2^a,b^ (%)	*n*	<–2/<–1/>1/>2 (%)	*n*	<–2/<–1/>1/>2 (%)	*n*	<–2/<–1/>1/>2 (%)
Birth	6 weeks	1109	846	5.9/22.7/14.3/2.4	1100	9.9/36.2/5.4/0.4	840	25.1/46.7/14.5/5.7	977	2.1/10.0/33.1/10.1
6 weeks	3 months	1065	796	0.3/7.2/13.9/1.3	1048	0.7/8.3/6.1/0.6	792	5.6/23.1/14.8/3.3	921	0.2/6.2/4.2/0.4
3 months	6 months	1033	977	0.6/7.9/18.0/1.9	1006	0.1/3.7/13.3/2.1	972	1.0/7.6/26.7/6.4	965	0.2/3.3/10.4/0.4
6 months	12 months	1023	977	2.4/18.3/7.2/1.1	1001	0.1/6.2/14.1/1.5	973	0.6/6.6/24.8/5.4	945	0.2/5.7/11.2/0.3
12 months	15 months	1010	977	0.2/7.7/5.3/0.3	995	0.0/1.5/2.3/0.1	966	0.3/3.9/8.6/0.3	–	–
15 months	24 months	1041	1000	2.2/23.0/4.5/0.5	1024	0.5/9.9/4.1/0.2	985	0.9/11.7/20.5/3.4	–	–
24 months	4 years	1244	1211	0.6/8.3/6.3/0.3	1217	0.5/10.8/4.8/0.5	1049	3.2/20.0/11.3/2.4	–	–

* Head circumference is systematically recorded up to 12 months of age.

a <–2: crossing two or more percentile channels down (change in *z*-score ⩽–1.34); <–1: crossing one or more percentile channel down (change in *z*-score ⩽–0.67; also includes two or more channels down); >2: crossing two or more percentile channels up (change in *z*-score ⩾1.34); >1: crossing one or more percentile up (change in *z*-score ⩾0.67; includes crossing two or more channels up).

b Elaborate tables with all age combinations are presented in Supplemental Table SI.

Using the BGS1 reference (Table III), when excluding changes between birth and six weeks, crossing centile channels was more symmetrical concerning upward and downward crossing for head circumference. In total, 36% of the children crossed one or more channels up/down for head circumference between six weeks and one year of age (Supplemental Table SI).

### Follow-up and referral according to the guidelines

A total of 37.9% of the children were identified for short length/downward crossing, 8.8% for low weight/downward crossing, 13.6% for high weight-for-length or BMI and 33.0% for large head circumference for further follow-up or referral according to the guidelines from the Norwegian Directorate of Health (Table IV). The recommended guidelines for identifying children for further action are summarised in Supplemental Table SII.

**Table IV.** Number of children identified for further action (follow-up at the well-baby clinic or referral to specialist health care) following the national guidelines.

Age	Length^a^		Low weight^b^		High weight/BMI^c^		Head circumference^d^	
	*n*	Follow-up, *n* (%)	*n*	Follow-up, *n* (%)	*n*	Follow-up, *n* (%)	*n*	Follow-up, *n* (%)
6 weeks	846	7 (0.8)	1100	5 (0.4)	–	–	993	7 (0.7)
3 months	1109	3 (0.3)	1132	14 (1.2)	–	–	1093	40 (3.6)
6 months	1100	1 (0.1)	1106	23 (2.1)	1108	23 (2.1)	1057	157 (14.8)
12 months	1182	2 (0.2)	1168	31 (2.6)	1172	25 (2.1)	974	204 (20.9)
15 months	1108	4 (0.4)	1062	13 (1.2)	1075	21 (1.9)	–	–
24 months	1347	407 (30.2)	1302	41 (3.1)	1292	85 (6.6)	–	–
4 years	1155	199 (17.2)	–	–	1392	81 (5.8)	–	–
**Total** ^e^	**1540**	**584 (37.9)**	**1383**	**122 (8.8)**	**1517**	**206 (13.6)**	**1150**	**380 (33.0)**

Prevalence of children is presented for rules of follow-up for short length-for-age, low weight-for-age, high weight-for-length or BMI for age, and large head circumference-for-age.

a Length below the third percentile (–2SD) or crossing down more than one percentile channel over an interval of 12–18 months during the first two years or crossing down more than one percentile channel between two and five years of age.

b Weight below the third percentile (–2SD) or weight below third percentile and abnormal length-for-age or crossing down two percentile channels or more during the first two years of life.

c Weight-for-length above +2SD between six months and two years or a BMI on or above IOTF-25 between two and five years.

d Head circumference is ≥2.5SD or crosses more than one percentile channel up.

e The ‘total’ row indicates the number of children who attended the last visit (four years for length and high weight, 24 months for low weight and 12 months for head circumference) and were identified at any scheduled contact since birth.

Only 1.8% of the children were identified for a short length between 6 weeks and 15 months (Table IV). The number of children identified for short height was significantly larger at two and four years of age, which was almost exclusively due to the downward crossing of centile channels (342/407 identified at two years of age, and 178/199 identified at four years of age). At two years of age, 42/1363 (3.1%) children were identified for follow-up because of tall stature, and another 20/1500 (1.3%) were identified at four years of age. In total, 60/1540 (3.9%) children were identified because of tall stature (data not shown).

The frequency of children identified for a low weight was small (0.4%–3.1%) throughout the whole age range (Table IV). Low weight-for-age was assessed up to two years of age as recommended in the guidelines. The assessment of high weight-for-length was evaluated from six months of age, and the frequency was stable around 2% up to two years of age and about 6% for a high BMI.

Head circumference is systematically assessed during the first year of life in routine practice. Follow-up is recommended if head circumference crosses one percentile channel or more between any combination of ages, with referral to specialist health care if it measures +2.5SD or more at any age. The number of children identified as such was very high (up to 20%), which was found to be almost exclusively attributable to the evaluation of centile crossing (149/157 children at 6 months and 195/204 children at 12 months were identified due to crossing more than one percentile channel). The prevalence of a head circumference of +2.5SD or more was <1% at each age (data not shown). Children with a relatively small head circumference are referred only if the head circumference flattens from the third percentile, which was the case for only five children (<0.5%) in our sample.

## Discussion

This study evaluated the Norwegian guidelines for growth monitoring in children between birth and five years of age. By analysing a healthy population, we were able to map the proportion of children identified to estimate the clinical workload generated by the rules for follow-up and referral. The prevalence of children referred for concerns regarding actual ‘growth’, that is, crossing one or two channels up or down, was relatively high, suggesting the need for adjustment of the current guidelines for growth monitoring in Norway to prevent unnecessary check-ups or referrals to specialist health care and associated parental concerns.

The prevalence below the lower limit of −2SD was <2.3%, and the prevalence above the upper limit of +2SD was consequently >2.3%, confirming previous findings in children from Norway and other countries [2,22]. In Norway, this difference was found to be of clinical relevance in case of head circumference, leading to the acceptance of using local national charts when monitoring head circumference [23]. We expected a reasonable correspondence with the national growth reference, since the children in the present analysis are an integral part of the BGS1 reference sample. However, it is also important to note that the routine measurements analysed in this paper were extracted from an EHR in 2011 and were not used to construct the Norwegian reference chart in 2009. These routine measurements involved many different primary health-care nurses presenting daily clinical practice, contrary to the national growth reference study which involved few observers using strict measurement protocol and standardised equipment.

Channel crossing occurred much more frequently when measurements were compared to size at birth (e.g. between birth and six weeks of age), but these should be interpreted with caution because measurements at birth, in particular length and head circumference, are usually less reliable. In addition, channel crossing between consecutive target ages should be compared with caution because the time interval is variable (3–24 months). We have not stratified the measurements according to birth weight, as children with a high birth weight will most likely show a downward centile crossing towards their genetic potential and vice versa [11]. Mei et al. [7] analysed crossing of major percentile lines over six-month intervals from 0 to 60 months using the Centers for Disease Control and Prevention (CDC) growth charts and confirmed similar findings that the prevalence of percentile crossing for length and weight was most common during the first two years of life. However, they reported a higher prevalence of upward and downward crossing. In our data, 17% and 20% crossed two percentile channels or more for length and weight, respectively, between birth and six months, which is approximately half of the 31.9% and 38.8%, respectively, reported by Mei et al. [7]. Also, between six months and two years of age, the trends of crossing one and two percentile channels were much lower. Although we do not have the data, this might reflect differences in genetic or environmental factors affecting the dynamics of growth. Furthermore, the differences could be due to analysing methods and chart format with unequal bandwidth spacing.

The prevalence of upward and downward centile crossing presented in our analysis is a valuable addition to the literature, since studies on this are scarce, especially in European populations [7,24]. In this study, approximately 40% crossed one or more channels during the first year for all traits. In principle, these data can be helpful when devising guidelines by providing necessary estimates of channel crossing over time. Likewise, they can help health-care workers evaluate longitudinal growth trajectories in clinical practice.

The practice of growth monitoring and the use of clinical decision rules differ internationally, and optimally there should be a standardised practice with validated evidence-based tools [25]. Numerous prenatal risk factors could potentially influence birth weight, postnatal growth and long-term child health outcomes [26,27]. The guidelines are general and apply for healthy newborns (preterm infants are corrected to term age). Therefore, prenatal factors such as smoking, alcohol, gestational diabetes and socio-economic status are not identified in this paper. Although recommendations to follow up children at the well-baby clinic do not always lead to referral, it might lead to unnecessary worries or anxiety in parents and create an additional workload for health-care workers because of the required extra follow-up. Likewise, the confidence of the health-care workers may falter if too many children are detected as possibly worrisome. Although access to routine data was limited to 1806 healthy children, it consisted of 10,500 measurement contacts and was sufficient to estimate the clinical workload generated by the rules. Our analysis could be a good starting point to devise realistic and practically feasible rules for further evaluation or referral to specialist health care, in particular the high number of children being identified due to downward centile crossing for length from two years of age onwards, mostly detected by the dynamic rules. The criteria for follow-up concerning head circumference during the first year also need attention.

An optimal growth-monitoring programme requires evidence-based screening rules, adequate growth reference for the population and well-defined referral criteria to specialist health care [28–30]. Which growth chart will be better for growth monitoring is not obvious. Certain rules such as percentile crossing may increase the sensitivity to detect abnormal growth, but our results clearly show that such rules come at a cost, as they may inflate the follow-up rate. A cross-sectional reference chart gives no indication of how often this occurs, but our analysis of centile crossing might give some guidance on the expected prevalence.

Our study has some limitations. First, we only have anthropometric data from the routine follow-up in primary care and lack details about the clinical assessment. Significant clinical judgement, including anamnesis on pregnancy details, feeding practices, parental anthropometry and findings using both weight and length charts simultaneously, plays a substantial role when physicians refer to the specialist. Second, we cannot exclude participation bias in the current study, with 64% of the parents consenting to the retrieval of routine measurements from the well-baby clinics. The longitudinal data retrieved from the well-baby clinics are measurements taken by health-care professionals during scheduled routine contacts. It is known that the measurement variability in such settings is larger compared to a strict research environment involving few trained observers, but it is usually well within acceptable limits [19]. However, research data might not be representative of daily clinical practice, while the routinely collected data in our analysis represent precisely that. Despite these limitations, we believe that our data provide an important insight on current practice for growth monitoring during the first five years of life.

## Conclusions

In general, the guidelines criteria resulted in a relatively large proportion of healthy children identified for further follow-up or referral. When using the recommended guidelines, we observed a higher than expected number above +2SD and a lower number below −2SD. Despite this, most children were in fact identified by the existing rules for changes in growth over time, that is, crossing of percentile channels. A revision of the Norwegian guidelines is advisable, as two out of five healthy children were identified for downward crossing of length and one in three were identified by the recommended rules for monitoring head circumference.

## Supplemental Material

sj-docx-1-sjp-10.1177_14034948231187513 – Supplemental material for Evaluating national guidelines for monitoring early growth using routinely collected data in Bergen, Norway Supplemental material, sj-docx-1-sjp-10.1177_14034948231187513 for Evaluating national guidelines for monitoring early growth using routinely collected data in Bergen, Norway by Melissa R. Balthasar, Mathieu Roelants, Bente Brannsether-Ellingsen, Kristine M. Stangenes, Maria C. Magnus, Siri E. Håberg, Simon N. Øverland and Pétur B. Júlíusson in Scandinavian Journal of Public Health
