# Supplementary material for: Evaluating national guidelines for monitoring early growth using routinely collected data in Bergen, Norway
Source: Scand J Public Health. 2023 Jul 27;52(6):718–25. doi: 10.1177/14034948231187513 (PMC11308290; doi:10.1177/14034948231187513)
Supplement: sj-docx-1-sjp-10.1177_14034948231187513 – Supplemental material for Evaluating national guidelines for monitoring early growth using routinely collected data in Bergen, Norway [file sj-docx-1-sjp-10.1177_14034948231187513.docx]

**Evaluating national guidelines for monitoring early growth, using routinely collected data in Bergen, Norway.**

Melissa R. Balthasar, Mathieu Roelants, Bente Brannsether-Ellingsen, Kristine M. Stangenes, Maria C. Magnus, Siri E. Håberg, Simon N. Øverland, Pétur B. Júlíusson

Scandinavian Journal of Public Health, 2023

**Supplementary data**

**Supplementary Figure 1.** Flow chart showing selection of children eligible for the present analysis. **
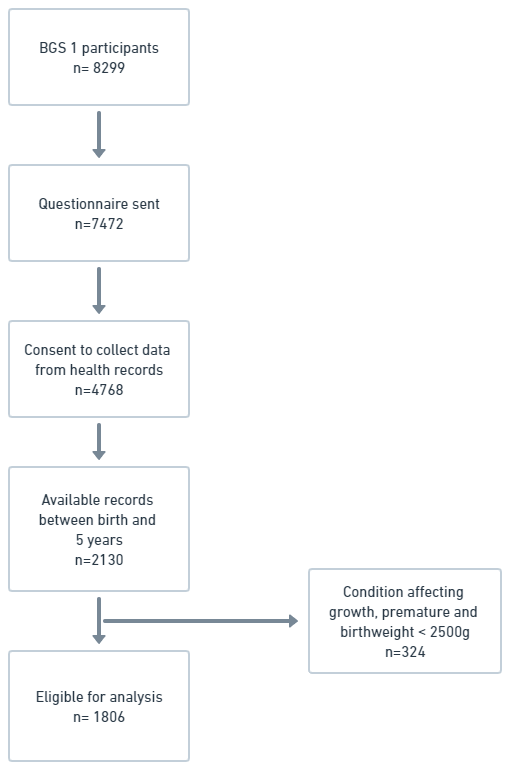
**

**Supplementary Figure 2.** Identification and selection of age intervals around the scheduled contacts at the ages of 6 weeks (4 – 10 weeks, n = 1109), 3 months (2.5 – 4 months, n = 1147), 6 months (5.5 – 7 months, n = 1140), 12 months (11.5 – 14.5 months, n = 1218), 15 months (14.5 – 17.5 months, n =1141), 24 months (23 – 36 months, n =1396, and 4 years (3.75 – 5 years, n = 1547). The graph shows all available contacts per week, and contacts selected for analysis. The precise age range and number of selected contacts is given between brackets. When children were measured twice within a target range, the nearest contact was selected.

**
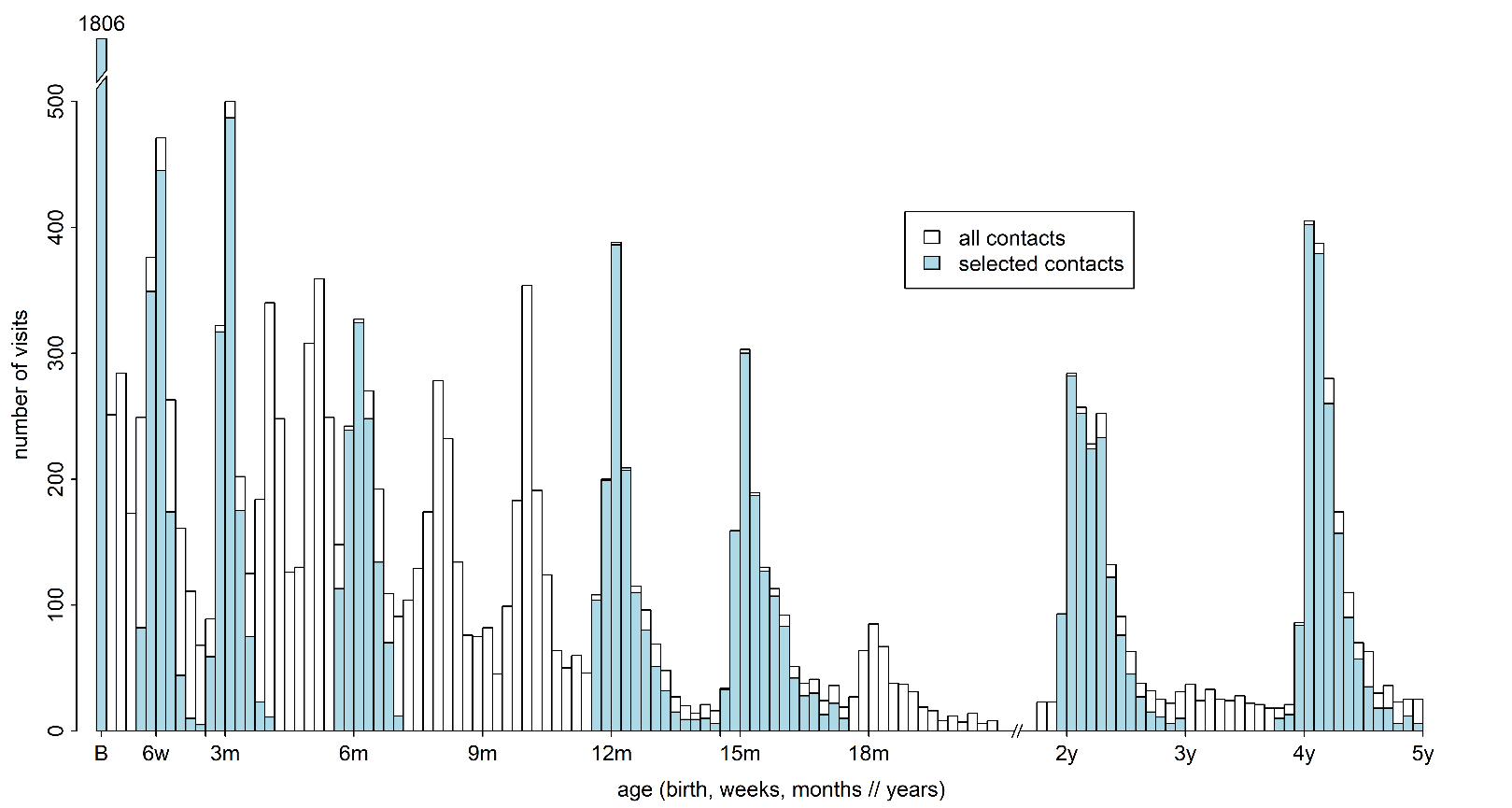
**

**Supplementary figure 3.** Prevalence of children crossing one and two percentile channels for length/height, weight, weight-for-length and head circumference. Comparing to WHO growth standards for length/height and weight, and to national growth reference (BGS) for head circumference are part of the guideline. White bar indicates children who did not cross any percentile channel(s), green bar indicates crossing one channel down, yellow bar crossing two or more channels down, red bar crossing one channel up, and blue bar crossing two or more channels up. B= birth, 6w= 6 weeks, 3m= 3months, 6m=6months, 12m=12months, 15m=15months, 24m= 24 months, 4y=4years.


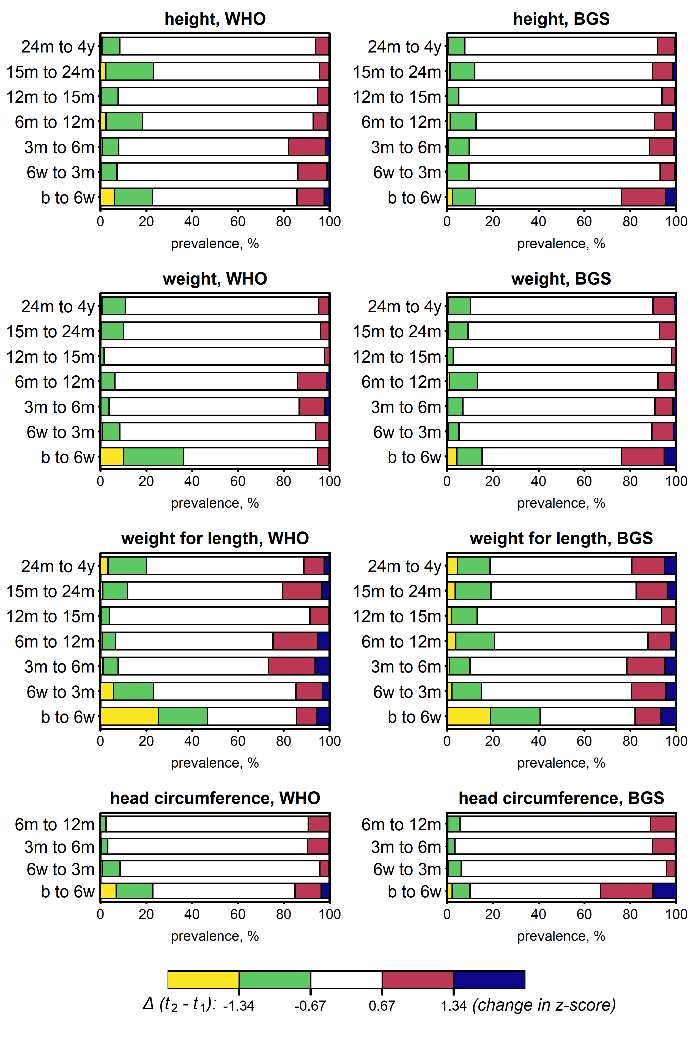


**Supplementary table 1.** Crossing of centile channels for length (A), weight (B), weight for length (C), and head circumference (D) between all age combinations available from birth to 5 years of age. Each table shows the number of observation pairs (first line), the prevalence of crossing one or more (Δz ≤ -0.67) and two or more (Δz ≤ -1.34) channels down (second line) and one or more (Δz ≥ 0.67) and two or more (Δz ≥1.34) channels up. Note that the prevalence of crossing one or more channels also includes the children who cross two or more channels according to this definition. The prevalence when using the WHO growth standards is given above the diagonal, the prevalence when using the BGS reference below the diagonal.

**A. Length for age**

| N  ≤ -0.67; ≤ -1.34  ≥ 0.67; ≥ 1.34 | birth | 6 weeks | 3 months | 6 months | 12 months | 15 months | 24 months | 4 years |
| --- | --- | --- | --- | --- | --- | --- | --- | --- |
| Birth | WHO  **BIRTH**  BGS | n=846  22.7; 5.9  14.3; 2.4 | n=1114  20.9; 5.2  20.6; 5.0 | n=1107  20.8; 5.9  25.6; 11.0 | n=1185  29.2; 11.2  23.8; 10.0 | n=1116  32.3; 13.5  21.0; 8.5 | n=1353  41.5; 19.4  14.9; 6.3 | n=1530  42.4; 20.8  14.5; 5.9 |
| 6 weeks | n=846  12.4; 2.2  24.0; 4.7 | WHO  **6 WEEKS**  BGS | n=796  7.2; 0.3  13.9; 1.3 | n=746  11.3; 0.5  24.7; 6.2 | n=748  20.6; 4.5  22.2; 7.9 | n=705  22.8; 6.7  20.4; 5.7 | n=741  36.8; 14.7  13.5; 5.0 | n=703  36.8; 14.8  15.9; 4.1 |
| 3 months | n=1114  14.4; 3.1  25.9; 5.6 | n=796  9.5; 0.1  7.0; 0.5 | WHO  **3 MONTHS**  BGS | n=977  7.9; 0.6  18.0; 1.9 | n=992  20.8; 2.9  16.3; 4.2 | n=924  25.5; 6.4  15.2; 3.7 | n=969  39.7; 15.3  11.2; 2.9 | n=925  41.5; 13.8  12.8; 3.6 |
| 6 months | n=1107  18.2; 3.8  26.3; 9.5 | n=746  17.3; 1.6  14.2; 2.7 | n=977  9.6; 0.6  11.8; 0.9 | WHO  **6 MONTHS**  BGS | n=977  18.3; 2.4  7.2; 1.1 | n=897  24.6; 4.6  7.4; 0.9 | n=953  40.9; 13.3  4.7; 0.7 | n=916  40.8; 13.3  5.5; 1.0 |
| 12 months | n=1185  23.2; 7.0  27.3; 11.0 | n=748  24.6; 5.2  17.0; 5.5 | n=992  19.8; 2.4  15.4; 3.3 | n=977  12.6; 1.3  9.5; 1.3 | WHO  **12 MONTHS**  BGS | n=977  7.7; 0.2  5.3; 0.3 | n=1050  29.1; 4.2  4.5; 0.7 | n=1011  31.6; 6.1  6.9; 0.5 |
| 15 months | n=1116  22.8; 8.3  27.5; 9.9 | n=705  24.0; 6.4  19.1; 5.7 | n=924  19.3; 4.7  16.7; 3.8 | n=897  16.4; 2.2  11.1; 1.6 | n=977  5.0; 0.1  6.2; 0.5 | WHO  **15 MONTHS**  BGS | n=1000  23.0; 2.2  4.5; 0.5 | n=965  25.5; 3.2  5.3; 0.2 |
| 24 months | n=1353  25.4; 9.5  26.2; 11.2 | n=741  28.9; 10.0  18.5; 7.0 | n=969  26.2; 7.1  18.6; 6.4 | n=953  20.5; 4.6  15.0; 2.7 | n=1050  12.4; 1.5  12.9; 2.1 | n=1000  12.0; 1.2  10.4; 1.4 | WHO  **2 YEARS**  BGS | n=1211  8.3; 0.6  6.3; 0.3 |
| 4 years | n=1530  25.1; 10.0  27.8; 11.2 | n=703  28.2; 8.8  21.1; 8.1 | n=925  24.8; 7.2  20.8; 7.5 | n=916  21.1; 5.6  18.3; 2.4 | n=1011  15.7; 2.4  16.4; 2.5 | n=965  14.0; 1.7  14.2; 1.1 | n=1211  7.7; 0.7  8.2; 0.5 | WHO  **4 YEARS**  BGS |

**B. Weight for age**

| N  ≤ -0.67; ≤ -1.34  ≥ 0.67; ≥ 1.34 | Birth | 6 weeks | 3 months | 6 months | 12 months | 15 months | 24 months | 4 years |
| --- | --- | --- | --- | --- | --- | --- | --- | --- |
| Birth | WHO  **BIRTH**  BGS | n=1100  36.2; 9.9  5.4; 0.4 | n=1136  42.1; 19.2  9.5; 1.6 | n=1122  39.3; 16.0  15.2; 4.1 | n=1207  35.5; 14.5  17.2; 6.3 | n=1130  34.6; 14.2  17.3; 6.0 | n=1382  36.4; 15.9  17.0; 5.6 | n=1523  42.8; 18.0  13.2; 5.2 |
| 6 weeks | n=1100  15.3; 4.2  23.8; 5.2 | WHO  **6 WEEKS**  BGS | n=1048  8.3; 0.7  6.1; 0.6 | n=983  16.7; 2.3  20.1; 5.5 | n=986  16.3; 2.9  24.9; 9.9 | n=916  14.3; 2.7  26.4; 10.3 | n=968  17.8; 3.4  25.2; 9.1 | n=905  23.8; 5.5  21.3; 7.4 |
| 3 months | n=1136  21.5; 6.5  30.5; 10.6 | n=1048  5.2; 0.4  10.7; 1.1 | WHO  **3 MONTHS**  BGS | n=1006  3.7; 0.1  13.3; 2.1 | n=1019  8.7; 0.7  26.9; 7.8 | n=945  8.8; 1.2  27.8; 8.5 | n=1001  12.5; 1.9  25.5; 8.6 | n=936  17.6; 3.5  22.2; 7.5 |
| 6 months | n=1122  22.6; 7.8  31.6; 13.8 | n=983  17.0; 2.6  19.8; 6.0 | n=1006  6.9; 0.2  9.3; 1.4 | WHO  **6 MONTHS**  BGS | n=1001  6.2; 0.1  14.1; 1.5 | n=918  8.4; 0.8  17.3; 2.4 | n=980  14.2; 2.1  18.2; 3.9 | n=924  19.2; 4.3  16.6; 3.2 |
| 12 months | n=1207  26.0; 10.4  28.3; 11.6 | n=986  25.1; 6.4  21.1; 8.2 | n=1019  21.0; 2.6  16.1; 4.6 | n=1001  13.2; 1.0  8.0; 0.8 | WHO  **12 MONTHS**  BGS | n=995  1.5; 0.0  2.3; 0.1 | n=1078  11.5; 0.8  9.1; 0.6 | n=1015  19.7; 3.3  9.9; 1.0 |
| 15 months | n=1130  26.3; 11.0  28.3; 10.9 | n=916  25.7; 6.8  20.1; 8.1 | n=945  21.4; 4.7  16.0; 4.3 | n=918  15.4; 2.1  10.0; 1.6 | n=995  2.6; 0.0  2.1; 0.1 | WHO  **15 MONTHS**  BGS | n=1024  9.9; 0.5  4.1; 0.2 | n=969  19.5; 1.8  6.7; 0.2 |
| 24 months | n=1382  27.1; 11.1  27.9; 11.8 | n=968  28.2; 8.5  18.9; 8.2 | n=1001  26.4; 5.9  16.4; 5.5 | n=980  21.9; 4.7  13.8; 3.2 | n=1078  13.5; 1.6  11.1; 0.9 | n=1024  9.1; 0.4  7.3; 0.3 | WHO  **2 YEARS**  BGS | n=1217  10.8; 0.5  4.8; 0.5 |
| 4 years | n=1523  28.8; 12.3  26.2; 11.4 | n=905  30.6; 10.3  20.6; 7.8 | n=936  26.5; 7.6  17.5; 6.4 | n=924  25.0; 6.5  14.1; 3.7 | n=1015  16.9; 3.2  14.9; 2.9 | n=969  16.4; 1.8  12.5; 1.4 | n=1217  10.3; 0.3  10.1; 0.7 | WHO  **4 YEARS**  BGS |

**C. Weight for length**

| N  ≤ -0.67; ≤ -1.34  ≥ 0.67; ≥ 1.34 | Birth | 6 weeks | 3 months | 6 months | 12 months | 15 months | 24 months | 4 years |
| --- | --- | --- | --- | --- | --- | --- | --- | --- |
| Birth | WHO  **BIRTH**  BGS | n=840  46.7; 25.1  14.5; 5.7 | n=1109  50.1; 29.3  14.2; 5.3 | n=1102  41.6; 21.2  20.0; 7.9 | n=1180  33.9; 15.1  27.2; 11.8 | n=1104  30.3; 13.8  28.0; 12.2 | n=1338  25.9; 12.8  31.8; 15.8 | n=1320  30.1; 14.0  26.7; 11.1 |
| 6 weeks | n=842  40.6; 19.0  17.9; 6.9 | WHO  **6 WEEKS**  BGS | n=792  23.1; 5.6  14.8; 3.3 | n=740  19.2; 7.3  29.9; 11.8 | n=743  17.9; 5.5  39.6; 19.1 | n=697  13.9; 5.6  42.5; 23.1 | n=731  12.6; 4.2  46.1; 25.7 | n=623  14.9; 4.5  39.6; 20.2 |
| 3 months | n=1111  39.6; 20.3  21.4; 8.9 | n=792  15.0; 2.0  19.6; 4.5 | WHO  **3 MONTHS**  BGS | n=972  7.6; 1.0  26.7; 6.4 | n=987  6.9; 1.2  43.1; 18.1 | n=914  6.8; 1.4  46.7; 21.4 | n=959  6.4; 1.5  53.5; 25.9 | n=799  6.6; 1.8  48.3; 22.2 |
| 6 months | n=1104  33.6; 17.8  24.8; 11.7 | n=740  15.7; 4.6  29.6; 12.0 | n=972  10.0; 1.1  21.6; 4.8 | WHO  **6 MONTHS**  BGS | n=973  6.6; 0.6  24.8; 5.4 | n=887  7.1; 1.0  31.7; 9.8 | n=944  8.1; 1.5  41.7; 14.6 | n=797  11.2; 3.0  35.9; 13.2 |
| 12 months | n=1182  38.5; 21.0  22.1; 10.2 | n=743  27.3; 10.9  25.0; 9.3 | n=987  21.3; 5.6  22.3; 7.1 | n=973  20.7; 3.7  12.3; 2.5 | WHO  **12 MONTHS**  BGS | n=966  3.9; 0.3  8.6; 0.3 | n=1040  9.0; 1.1  26.2; 6.2 | n=884  13.3; 2.5  25.3; 5.8 |
| 15 months | n=1106  42.0; 23.1  19.7; 8.5 | n=697  25.7; 10.9  25.4; 11.5 | n=914  25.4; 7.7  21.1; 6.0 | n=887  27.5; 6.9  12.4; 2.5 | n=966  13.0; 1.9  6.4; 0.1 | WHO  **15 MONTHS**  BGS | n=985  11.7; 0.9  20.5; 3.4 | n=845  16.0; 2.6  20.8; 4.5 |
| 24 months | n=1341  40.5; 23.5  21.0; 9.5 | n=731  29.3; 13.8  25.0; 11.4 | n=959  27.9; 10.4  21.4; 8.1 | n=944  32.1; 12.4  15.7; 4.8 | n=1040  23.8; 5.8  16.6; 3.4 | n=985  19.1; 3.5  17.5; 4.0 | WHO  **2 YEARS**  BGS | n=1049  20.0; 3.2  11.3; 2.4 |
| 4 years | n=1506  41.6; 24.3  20.6; 9.9 | n=697  29.6; 12.9  26.3; 13.3 | n=915  28.9; 9.4  23.9; 8.3 | n=907  31.4; 11.8  17.2; 5.0 | n=1000  24.9; 6.8  18.9; 5.6 | n=949  20.0; 4.8  22.1; 6.4 | n=1186  18.8; 4.6  19.4; 5.1 | WHO  **4 YEARS**  BGS |

**D. Head circumference**

| N  ≤ -0.67; ≤ -1.34  ≥ 0.67; ≥ 1.34 | Birth | 6 weeks | 3 months | 6 months | 12 months |
| --- | --- | --- | --- | --- | --- |
| Birth | WHO  **BIRTH**  BGS | n=977  22.8; 6.8  15.1; 3.7 | n=1079  30.9; 9.7  14.7; 4.5 | n=1074  28.4; 10.4  20.2; 7.3 | n=1093  24.2; 10.3  26.5; 10.9 |
| 6 weeks | n=977  10.0; 2.1  33.1; 10.1 | WHO  **6 WEEKS**  BGS | n=921  8.5; 0.7  4.3; 0.4 | n=875  10.2; 0.7  13.3; 2.6 | n=856  10.0; 1.4  22.4; 5.5 |
| 3 months | n=1079  14.4; 3.2  33.2; 10.4 | n=921  6.2; 0.3  4.2; 0.4 | WHO  **3 MONTHS**  BGS | n=965  3.1; 0.2  9.7; 0.3 | n=958  4.3; 0.1  21.9; 2.6 |
| 6 months | n=1074  15.5; 3.9  38.2; 15.5 | n=875  10.7; 0.6  14.5; 2.5 | n=965  3.3; 0.2  10.4; 0.4 | WHO  **6 MONTHS**  BGS | n=945  2.3; 0.0  9.3; 0.1 |
| 12 months | n=1093  16.8; 5.9  42.6; 20.4 | n=856  13.6; 2.7  22.8; 6.3 | n=958  8.0; 0.9  22.4; 3.5 | n=945  5.7; 0.2  11.2; 0.3 | WHO  **12 MONTHS**  BGS |

**Supplementary Table 2**. Criteria for follow up and referral in the current Norwegian guidelines for measurements of length, weight and head circumference (clinical evaluation not included in this table).

| **Rule for follow up or referral** | **Age range** | **Description** |
| --- | --- | --- |
| I | Birth- 4 weeks | Follow up if infant looses more than 10% of their birthweight, or failure to obtain birthweight within 14 days. |
| II | Birth- 4 weeks | Refer if failure to obtain birth weight after 21 days (3 weeks). |
| III | 4 weeks- 2 years | Follow up if length-for-age is below the 3^rd^ percentile. |
| IV | 4 weeks- 2 years | In the case of a downward crossing of one percentile channel for length after 12-18 months of age, the child is followed up with annual measurements until the growth has been clarified. |
| V | 2-5 years | Follow up if length-for-age is on or below the 3^rd^ percentile, and if further measurements shows growth deceleration, the child should be referred. |
| VI | - 1. years | Follow up if length is on or above the 97^th^ percentile, and if further measurements shows increasing growth, the child should be referred. |
| VII | - 1. years | Follow up when the child shows faltering growth between two measurements less than 2 percentile channels. |
| VIII | 2-5 years | When crossing two percentile channels for length between two measurements, consider referral. |
| IX | 4 weeks- 2 years | Follow up if weight-for-age is below the 3^rd^ percentile. |
| X | 4 weeks- 2 years | Follow up if weight-for-age crosses down more than two percentile channels. |
| XI | 4 weeks- 2 years | Refer if weight-for-age is below the 3^rd^ percentile in addition deviant length-for-age. |
| XII | 4 weeks- 2 years | Follow up if weight-for-length is above the 97^th^ percentile (does not apply to children who are exclusively breastfed up to 6 months of age). |
| XIII | 2- 5years | Follow up if the BMI is on or above IOTF-25 . |
| XIV | 2-5years | Refer if the BMI is on or above IOTF-30. |
| XV | 7 (10) days - 18months | Refer if head circumference is at or above +2.5 SD. |
| XVI | 7 (10) days - 18months | Follow up if crossing of head circumference 1 or up to 2 percentile channels without clinical findings. |
| XVII | 7 (10) days - 18months | When crossing more than 2 percentile channels up without clinical findings, the measurement is repeated within 4 weeks. If further increase the child is referred. |
| XVIII | 7 (10) days - 18months | Children with small heads are referred only if the head circumference flattens from the 3rd percentile. |
